# One‐Step MOF‐Templated Strategy to Fabrication of Ce‐Doped ZnIn_2_S_4_ Tetrakaidecahedron Hollow Nanocages as an Efficient Photocatalyst for Hydrogen Evolution

**DOI:** 10.1002/advs.202104579

**Published:** 2022-01-09

**Authors:** Huitao Fan, Yujie Jin, Kecheng Liu, Weisheng Liu

**Affiliations:** ^1^ Key Laboratory of Nonferrous Metal Chemistry and Resources Utilization of Gansu Province and State Key Laboratory of Applied Organic Chemistry College of Chemistry and Chemical Engineering Lanzhou University Lanzhou 730000 P. R. China; ^2^ College of Chemistry and Pharmaceutical Engineering Nanyang Normal University Nanyang 473061 P. R. China

**Keywords:** Ce‐metal–organic frameworks, Ce‐doped ZIS, photocatalytic hydrogen evolution, tetrakaidecahedron nanocages, ZnIn_2_S_4_

## Abstract

Achieving structure optimizing and component regulation simultaneously in the ZnIn_2_S_4_‐based photocatalytic system is an enormous challenge in improving its hydrogen evolution performance. 3D hollow‐structured photocatalysts have been intensively studied due to their obvious advantages in solar energy conversion reactions. The synthesis of 3D hollow‐structured ZnIn_2_S_4_, however, is limited by the lack of suitable template or synthesis methods, thereby restricting the wide application of ZnIn_2_S_4_ in the field of photocatalysis. Herein, Ce‐doped ZnIn_2_S_4_ photocatalysts with hollow nanocages are obtained via one‐step hydrothermal method with an ordered large‐pore tetrakaidecahedron cerium‐based metal–organic frameworks (Ce‐MOFs) as template and Ce ion source. The doping of Ce and the formation of ZnIn_2_S_4_ tetrakaidecahedron hollow nanocages with ultrathin nanosheet subunits are simultaneously induced by the Ce‐MOFs, making this groundbreaking work. The Ce‐doped ZnIn_2_S_4_ with a nonspherical 3D hollow nanostructure inherit the tetrakaidecahedron shape of the Ce‐MOF templates, and the shell is composed of ultrathin nanosheet subunits. Both theoretical and experimental results indicate that the doping of Ce and the formation of hollow nanocages increase light capture and the separation of photogenerated charge carriers.

## Graphical abstract

Ce‐doped ZnIn_2_S_4_ photocatalysts with hollow nanocages are obtained via one‐step hydrothermal method with an ordered large‐pore tetrakaidecahedron cerium‐based metal–organic frameworks as template and Ce ion source.

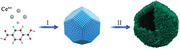

## 1 Introduction

Replacing traditional fossil fuels with renewable and sustainable energy sources is an important strategy to cope with the fast‐growing energy demand and increasing environmental pollution.^[^ ^1^, ^2^, ^3^, ^4^ ^]^ As a new renewable energy source, hydrogen energy is regarded as the most promising clean energy source due to its great potential to replace traditional fossil fuels.^[^ ^5^, ^6^, ^7^ ^]^ Photocatalytic water splitting for H_2_ evolution has been considered one of the most promising strategies to tackle the environmental and energy crisis.^[^ ^8^, ^9^, ^10^, ^11^ ^]^ To construct high‐performance photocatalytic systems for water splitting that meet the needs of practical applications, it is crucial to design photocatalysts with good stability, a low cost, and high activity.^[^ ^12^, ^13^, ^14^ ^]^ To date, a multitude of semiconductor photocatalysts have been explored to evaluate their potential for hydrogen production efficiency; unfortunately, most of them have low photocatalytic activity restricted by their weak adsorption of visible light, narrow solar light response range, and poor utilization of photon‐generated carriers.^[^ ^15^, ^16^, ^17^, ^18^, ^19^, ^20^, ^21^ ^]^

In recent years, transition metal sulfide photocatalysts have shown outstanding properties for water splitting because of their distinctive optical and electronic characteristics. Among them, ZnIn_2_S_4_ (ZIS) possesses a salient layered structure, a tunable bandgap (≈2.06–2.85 eV), a low toxicity and an excellent chemical stability.^[^ ^22^, ^23^, ^24^ ^]^ In addition, ultrathin 2D nanosheet structures of ZIS not only possess a high specific surface area but also shorten the diffusion distance of charge carriers to the abundance of exposed active sites.^[^ ^25^ ^]^ However, the excessive self‐assembly and aggregation of 2D ultrathin ZIS nanosheets greatly reduce its number of active sites and block photogenerated carrier separation, which severely restricts its photocatalytic activity.^[^ ^26^ ^]^ In general, pristine ZIS weakly adsorbs visible light; thus, it has a narrow solar light response range and poor charge separation/transfer efficiency.^[^ ^27^, ^28^ ^]^ To date, various strategies have been attempted to enhance the photocatalytic performance of ZIS. As previous reports, rationally regulating the chemical composition (e.g., elemental doping) of the photocatalyst can effectively expand its absorption of sunlight, accelerate charge transfer, and promote fast surface reaction kinetics.^[^ ^29^, ^30^ ^]^ For the past few years, various ions, such as N, O, P, Ni, Cu, Ag, have been doped into the ZIS to boost the photocatalytic activity. Benefiting from its 4f electronic transition characteristics and abundant energy levels, cerium, as a significant rare earth metal, has been widely reported in various fields including optical, electrical, and catalysis.^[^ ^31^ ^]^ Moreover, the doping of Ce has a great influence on optical properties that result in high charge mobility. More importantly, Ce possesses a Ce^3+^/Ce^4+^ variable feature, which can induce photochemical reactions and further improve the photocatalytic efficiency.^[^ ^32^, ^33^, ^34^ ^]^ Therefore, it will be excited to fabricate Ce‐doped ZIS to promote the separation of charge carriers and provide much more active sites for photocatalytic H_2_ generation. To the best of our knowledge, there has not yet been reported on Ce‐doped ZIS and its application for photocatalytic H_2_ evolution.

In addition to adjusting the chemical composition, the delicate structural design of catalysts plays a vital role in the realization of highly efficient photocatalytic reactions.^[^ ^35^ ^]^ In recent years, hollow‐structured photocatalysts have been intensively studied due to their obvious advantages in solar energy conversion reactions. The hollow‐structure can not only provide an abundance of active sites and a large surface area, but also shorten the distance for the transfer of charge carriers to boost photocatalytic reactions.^[^ ^36^, ^37^ ^]^ In addition, hollow structures can also enhance the light‐absorption capability by internal multilight scattering.^[^ ^38^ ^]^ Therefore, designing hollow micro‐ and nanostructures that grow in an array of ZnIn_2_S_4_ ultrathin nanosheet subunits as described above will inhibit aggregation and improve photocatalytic activity. Hard template method, acting as a classical strategy in the synthesis of hollow structured materials, has been widely used. However, to remove the template, specific conditions, such as high‐temperature annealing or etching processes in acid or alkali solutions, will be required, which may restrict their further applications of some materials that are not very stable in these circumstances. Therefore, finding a suitable template is the key factor in the synthesis of hollow ZIS. Metal–organic frameworks (MOFs), which can be etched away under relatively mild conditions, have attracted enormous research interest as templates to prepare hollow structured materials in recent years. However, for most MOFs, their microporous channels are small and etching takes a long time under mild conditions, which limits their application as templates for preparing hollow nanomaterials.

Herein, we demonstrate for the first time the rational design and construction of Ce‐doped ZIS tetrakaidecahedron hollow nanocages photocatalysts via one‐step hydrothermal method with an ordered large‐pore tetrakaidecahedron Ce‐MOFs as the template as well as the Ce ion source. In contrast to the traditional use of sacrificial templates for calcination or etching by strong acid and base, the ordered large‐pore Ce‐MOFs templates are tactfully degraded by thioacetamide (the raw material for synthesis of ZnIn_2_S_4_). Table S1 (Supporting Information) provides a comprehensive summary of the recently reported ZIS‐based photocatalysts with hollow structure prepared by different methods. The doping of Ce and the formation of ZIS tetrakaidecahedron hollow nanocages with ultrathin nanosheet subunits were induced simultaneously by the Ce‐MOFs, making this groundbreaking work. Both theoretical and experimental results indicate that, the synergistic effect of the nonspherical 3D hollow nanostructure and Ce doping greatly expand the absorption of sunlight, boost the separation and transport of charge carriers, provide a large surface area, and expose many more active sites for photocatalytic reactions. This unique photocatalyst, optimal Ce‐doped ZIS tetrakaidecahedron nanocages (ZTNs‐Ce20), exhibits improved photocatalytic hydrogen evolution activity (7.46 mmol h^−1^ g^−1^), which is much higher than that of pristine ZIS.

## 2 Results and Discussion

### 2.1 Characterization of Photocatalysts

**Figure 1:**
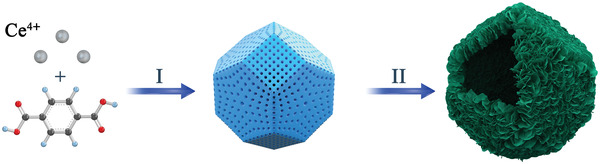
Schematic illustration of the synthetic process of a hierarchical ZTNs‐Ce20 tetrakaidecahedron hollow nanocages with ultrathin nanosheet subunits: (I) synthesis of an ordered large‐pore tetrakaidecahedron Ce‐MOFs, and (II) growth of ZIS nanosheets on the Ce‐MOFs through simultaneous etching and deposition and formation of ZTNs‐Ce20 tetrakaidecahedron hollow nanocages with ultrathin nanosheet subunits upon the complete template removal.

The synthesis routes for innovatively fabricating the intricate hollow architectures are schematically shown in **Figure** **1**. Starting with the synthesis of an ordered large‐pore tetrakaidecahedron Ce‐MOFs (step I), Ce‐doped ZIS hollow nanocage photocatalysts are built via a one‐step solvothermal method utilizing Ce‐MOFs as a template and cerium source (step II). During the reaction process, the Ce‐MOF templates are carefully degraded by thioacetamide (TAA). Finally, the perfect collaboration between Ce‐MOFs template degradation and ultrathin ZIS nanosheet growth leads to the formation of hollow ZTNs‐Cex nanocages. The Ce‐doped ZIS nanocages inherit the tetrakaidecahedron shape of the Ce‐MOF template, and the shell is composed of ultrathin nanosheet subunits. The mass percentage of Zn, In, Ce in ZTNs‐Cex were determined by the inductively coupled plasma (ICP) technique (Table S2, Supporting Information). As can be seen from Table S2 (Supporting Information), with the increase of Ce doping amount, the atom ratio of Zn:In decreases gradually, which fully indicates that Ce may replace Zn into the lattice of ZIS.

**Figure 2:**
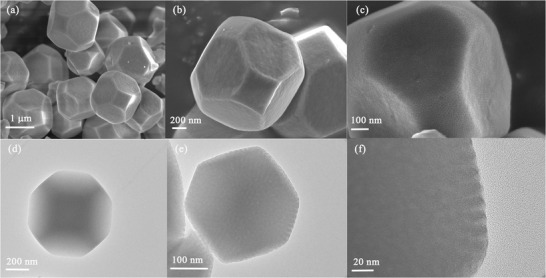
a–c) FESEM images and d–f) TEM image of Ce‐MOF tetrakaidecahedrons.

**Figure** **2** shows the morphology and structure of the as‐prepared Ce‐MOFs at different magnifications. The field‐emission scanning electronic microscopy (FESEM) images of the Ce‐MOFs show that the samples are regular tetrakaidecahedron with a size of ≈900 nm and have a uniform shape and uniformity of porous on the surface. Transmission electron microscopy (TEM) images indicate that these Ce‐MOFs are solid. At a higher magnification, the ordered array and uniformity of channels are clearly visible on the surface of the Ce‐MOF particles (Figure 2c,f). X‐ray diffraction (XRD) patterns further confirm the successful synthesis of the Ce‐MOFs (Figure S1, Supporting Information). As shown in Figure S2 (Supporting Information), the Brunauer–Emmett–Teller (BET) surface area of the Ce‐MOFs is 1036.5 m^2^ g^−1^. According to the Barrett–Joyner–Halenda (BJH) method, the corresponding pore size distribution curve indicates the mesoporosity of the Ce‐MOFs, and the pore size is centered at ≈9 nm.

**Figure 3:**
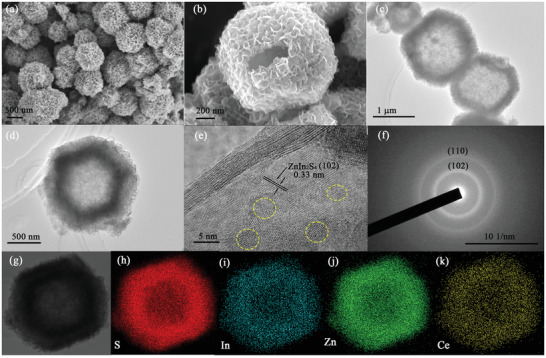
FESEM a,b), TEM c,d), and HRTEM e) images; corresponding SAED pattern f); and EDX elemental mapping g–k) images of the ZTNs‐Ce20 tetrakaidecahedron hollow nanocages.

Following, the as‐obtained tetrakaidecahedron Ce‐MOFs were utilized as a template and cerium source to fabricate Ce‐doped ZIS hollow tetrakaidecahedron nanocages through a one‐step solvothermal process. To obtain information about the appearance and morphology of the ZTNs‐Ce20 photocatalysts, the samples were examined by FESEM and TEM. From the FESEM images, it is observed that Ce‐doped ZTNs‐Ce20 still maintain the tetrakaidecahedron shape of the Ce‐MOFs, and the 3D structures of ZTNs‐Ce20 is fabricated with ultrathin nanosheet subunits, which makes the surface very rough (**Figure** **3**a,b). As seen from the partially broken samples in Figure 3b, the ZTNs‐Ce20 particles present a hollow tetrakaidecahedron morphology. The morphology and interior structure of the hollow ZTNs‐Ce20 nanocages were further investigated by TEM. The TEM image of the hollow ZTNs‐Ce20 nanocages shows that the hollow cages assembled by abundant nanosheets have a similar morphology and size as the Ce‐MOFs (Figure 3c,d). In the high‐resolution transmission electron microscopy (HRTEM) image of ZTNs‐Ce20, lattice fringes with an interplanar spacing of 0.33 nm correspond to the (102) facet of ZIS (Figure 3e), which is consistent with previous reports.^[^^35]^ Notably, crooked and discontinued crystal fringes are observed in the HRTEM image (indexed by the dotted circles in Figure 3e). These results show that there is plenty of structural distortion in the Ce‐doped ZIS nanosheets. The selected area electron diffraction (SAED) image (Figure 3f) presents two bright diffraction rings that can be indexed to the (102) and (110) planes of ZTNs‐Ce20. The elemental distributions of the ZTNs‐Ce20 hollow structure were determined by elemental mapping. Energy dispersive X‐ray detector (EDX) was used to obtain elemental mapping images (Figure 3g‐k ). These images indicate that S, In, Zn, and Ce are well distributed in ZTNs‐Ce20, further demonstrating that ZTNs‐Ce20 are successfully synthesized.

To deeply investigate the evolution mechanism of ZTNs‐Cex, mass‐dependent, and time‐dependent structural evolution experiments were performed, and the samples were analyzed by FESEM. The addition of Ce‐MOFs plays a vital role in constructing a tetrakaidecahedron hollow nanocage. As shown in Figure S3a,b (Supporting Information), when 10 and 15 mg of Ce‐MOFs are used, in addition to tetrakaidecahedron hollow nanocages, there are many self‐assembled microspheres. However, the content of self‐assembled microspheres gradually decreased and disappeared with the increasing of Ce‐MOFs. When the mass of Ce‐MOFs is 20 mg, the self‐assembled microspheres are hardly observed (Figure S3c, Supporting Information). Interestingly, with an increase of Ce‐MOFs, the Ce‐doped ZIS 2D nanosheets become thicker which may be due to the change in the crystal lattice caused by Ce ion doping (Figure S3d–f, Supporting Information). In addition, for comparison, ZIS nanoparticles were also synthesized under similar conditions. In the absence of Ce‐MOFs, only large‐scale self‐assembled microspheres could be found in the resultant product (Figure S4, Supporting Information). The time‐dependent structural evolution of ZTNs‐Ce20 was also monitored by FESEM, revealing the growth mechanism of ZIS nanosheet subunits. During the initial stage (10 min) of the solvothermal reaction, only a small amount of ZIS nanoparticles grow on the surface of Ce‐MOFs (Figure S5a, Supporting Information). This is mainly because Zn^2+^ and In^3+^ ions in the solution can coordinate with glycerol, which is adsorbed on the Ce‐MOF surface, to form complexes and then react with TAA to generate ZIS crystal nuclei on the Ce‐MOF surface. With the increasing of reaction time, the ZIS nanosheets continue to grow epitaxially and intensively accumulate (Figure S5b–d, Supporting Information). In the process of solvothermal reaction, the Ce‐MOF templates are degraded by TAA, and Ce ions are released. The Ce‐MOF@ZIS composite finally evolves into ZTNs‐Ce20 nanocages with ultrathin nanosheet subunits. According to the above analysis, the moderate additive mass of Ce‐MOFs (20 mg) favors the formation of ultrathin nanosheet subunits. As a consequence, ZTNs‐Cex hollow nanocages with ultrathin nanosheet subunits can be regulated by varying the template mass and reaction time.

During the synthesis process, the tetrakaidecahedron Ce‐MOFs with about 10 nm ordered mesoporous structure play an important role in the formation process of final ZTNs‐Ce. The particular tetrakaidecahedron Ce‐MOFs are conducive to the rapid entry of solvent molecules into the interior of MOFs, and etching of Ce‐MOFs from inside to outside under mild conditions, which is very favorable for the synthesis of hollow ZnIn_2_S_4_ in a short time (The synthesis time of ZnIn_2_S_4_ is generally 2 h). In order to determine whether the main factor during Ce‐MOFs degradation process is ligand protonation or the affinity of Ce^4+^ to S^2−^ released from TAA in the hydrothermal process, the Ce‐MOFs were immersed in hydrochloric acid (HCl) solutions (pH = 1, 3, 5, 7) and TAA solutions (30 mg TAA was dissolved in 20 mL aqueous solution with pH = 2.5) stirring at 80 °C for different times. SEM and TEM images were used to track the evolution of Ce‐MOFs. As shown in Figure S6 (Supporting Information), with the decrease of pH, the morphology of Ce‐MOFs is almost unchanged except that the surface becomes slightly rough after being soaked in HCl solution for 2 h, which fully indicated the Ce‐MOFs are stable under acidic conditions and protons have almost no effect on Ce‐MOFs degradation. However, the morphology of Ce‐MOF changes with different reaction time in TAA solution. As can be seen from the SEM images in Figure S7 (Supporting Information), the surface of Ce‐MOFs gradually becomes rough with the increase of reaction time. When the reaction time is 30 min, the broken Ce‐MOFs hollow structure can be observed, which fully proved that Ce‐MOFs etching carried out from inside to outside. This is supported by TEM images (Figure S8, Supporting Information) which demonstrate the internal structure changes of Ce‐MOFs in TAA solution at different time. As can be seen from Figure S8 (Supporting Information), with the increase of reaction time, the internal structure of Ce‐MOFs gradually becomes sparse, and then hollow structure appears, until the hollow structure completely collapses after reaction for 60 min. The reason for this phenomenon is that the inner core of MOFs has more defects than the outer shell, and the crystal outer layer is deemed to be chemically more robust than the inner layer.^[^ ^39^ ^]^ The results indicated that the affinity of Ce^4+^ to S^2−^ plays the main role during the Ce‐MOFs degradation. During the etching of Ce‐MOFs, S^2−^ captures Ce^4+^ to form Ce*_x_* S*_y_*. In fact, Ce*_x_* S*_y_* exists only as a transition state because they are unstable under acidic conditions (the pH value of synthesizing ZIS is 2.5).

**Figure 4:**
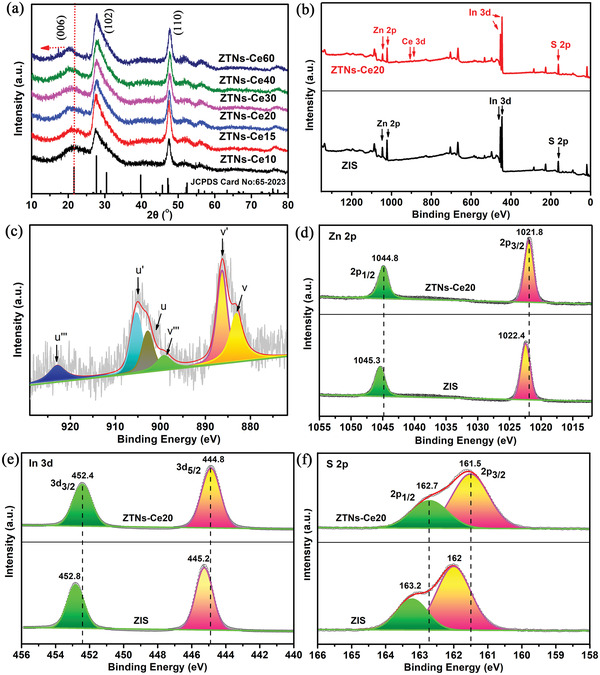
a) XRD pattern of ZTNs‐Cex (*x* = 10, 15, 20, 30, 40, 60), b) XPS survey spectra ZIS and ZTNs‐Ce20, c) High‐resolution XPS spectrum of Ce element in ZTNs‐Ce20, and high‐resolution XPS spectra of ZIS and ZTNs‐Ce20: d) Zn 2p, e) In 3d, and f) S 2p.

The crystal composition and structure of the as‐prepared ZTNs‐Cex were verified by their respective XRD patterns. The diffraction peaks of all samples are confirmed to be hexagonal ZIS (JCPDS card no. 65‐2023), demonstrating that the as‐obtained ZTNs‐Cex still maintain their original crystal structures (**Figure** **4**a). No other diffraction peaks (ZnS, In_2_S_3_, and CeOx) are detected, indicating the phase purity of ZTNs‐Cex. The results show that Ce doping has no significant effect on the crystallinity of product. However, it can be clearly seen that the peak corresponding to (006) shifts to lower angles with an increasing addition of Ce‐MOFs. This result suggests that Ce ions from the Ce‐MOFs might be successfully incorporated into the crystal lattice or substituted for the Zn or In in ZIS. Although Ce^4+^ and Ce^3+^ cannot easily replace Zn^2+^ or In^3+^ in the interior of the ZIS lattice because the ionic radii of Ce^4+^ (0.92 Å) and Ce^3+^ (1.03 Å) are larger than that of Zn^2+^ (0.74 Å) or In^3+^ (0.80 Å), with an increasing doping amount of Ce ions, some Ce^3+^ and Ce^4+^ still replace or insert into the lattice gap under the action of external force, leading to lattice distortion and resulting in a change in the crystal plane distance of ZIS. In order to identify the optimal form of atomic substitution for Ce in ZTNs, we calculated the formation energy of different substitution positions. As can be seen from Figure S9 (Supporting Information), when Ce replaces Zn, the formation energy value is lowest, compared with that of Ce substitute In or Ce enter into the lattice interlayer. Therefore, Ce tends to replace Zn into the ZTNs lattice, rather than randomly.

X‐ray photoelectron spectra (XPS) were used to examine the surface elemental compositions and identify the chemical states of the samples. As shown in Figure 4b, the survey spectra of the ZIS and ZTNs‐Ce20 samples verified the presence of Zn, In, and S, while the peak of Ce was observed only in the ZTNs‐Ce20 sample. The high‐resolution XPS spectra of Ce 3d for ZTNs‐Ce20 are displayed in Figure 4c. According to previous literature reports,^[^ ^40^, ^41^ ^]^ the peaks labeled u (900.6 eV) and u‴ (923.1 eV) correspond to Ce^4+^ 3d_3/2_, and the peaks marked v (882.4 eV) and v‴ (898.3eV) refer to Ce^4+^ 3d_5/2_. Additionally, the peaks marked as v′ (885.8 eV) and u′ (904.6 eV) are assigned to the Ce^3+^ species. Thus, the XPS measurement results reveal that two cerium species, namely, Ce^3+^ and Ce^4+^, are both present in ZTNs‐Ce20. The introduction of Ce^3+^ may result from the partial reduction of Ce^4+^ by H_2_S, which is generated by the hydrolysis of TAA.^[^ ^42^ ^]^ In the high‐resolution XPS spectrum of Zn 2p of ZTNs‐Ce20, the peaks located at 1044.8 and 1021.8 eV are assigned to Zn 2p_1/2_ and Zn 2p_3/2_ (Figure 4d), which indicates the Zn (II) oxidation state of ZTNs‐Ce20. The In 3d high‐resolution XPS spectrum shows two peaks at 444.8 eV (In 3d_5/2_) and 452.4 eV (In 3d_3/2_) (Figure 4e), corresponding to the In cation with a valence of +3. The S 2p peaks located at 162.7 and 161.5 eV are assigned to the S 2p_1/2_ and S 2p_3/2_ orbitals of the S^2−^ ions (Figure 4f).^[^^35]^ As seen from the high‐resolution XPS analysis, the Zn2p, In3d, and S2p peaks of ZTNs‐Ce20 obviously shift to lower binding energies than those of pristine ZIS (Figure 4d–f). These results further demonstrate the existence of the strong interactions between Ce and ZIS.^[^ ^43^ ^]^

**Figure 5:**
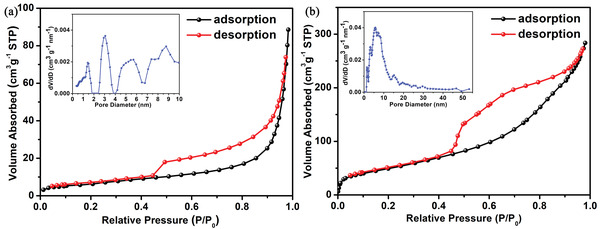
N_2_ sorption isotherms and the corresponding pore size distribution of a) ZIS and b) ZTNs‐Ce20.

The N_2_ adsorption and desorption experimental results show that both pure ZIS and ZTNs‐Ce20 exhibit a type IV isotherm (**Figure** **5**). Regarding ZIS and ZTNs‐Ce20, an obvious hysteresis loop can be observed, which indicates a mesoporous structure. The BET surface area of ZTNs‐Ce20 is185.67 m^2^ g^−1^, which is much larger than that of pure ZIS (22.52 m^2^ g^−1^). According to the BJH method, the corresponding pore size distribution curve indicates that the pore size of ZTNs‐Ce20 is centered at 8.4 nm, while pure ZIS is exhibit wide pore size distribution. It is speculated that the large surface area and pores in the nanosheet ZTNs‐Ce20 nanocages will facilitate mass transfer for heterogeneous catalysis.

### 2.2 Photocatalytic Activity

**Figure 6:**
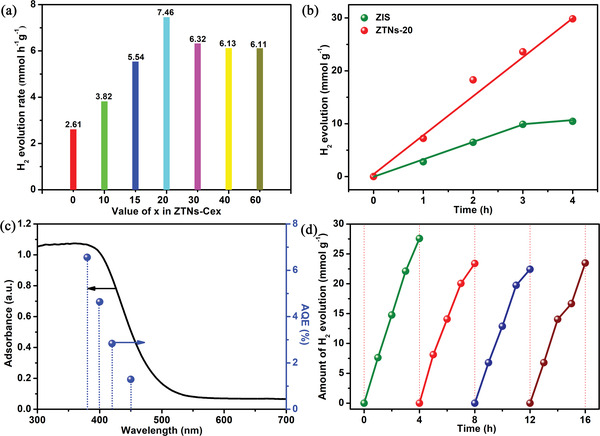
a) Photocatalytic H_2_ production activity of different samples after 4 h of light irradiation, b) time‐yield plots of H_2_, c) wavelength‐dependent AQE% with the ZTNs‐Ce20 catalyst under monochromatic light irradiation (380, 400, 420, 450 nm), and d) stability of ZTNs‐Ce20 for hydrogen production.

**Figure** **6**a depicts the photocatalytic hydrogen production efficiency of different samples under visible light irradiation (*λ* ≥ 400 nm). The nanosheet‐assembled ZIS particles possess a relatively low efficiency of H_2_ production (2.61 mmol g^−1^ h^−1^). Nonetheless, the efficiency of photocatalytic hydrogen production shows a normal distribution an increasing mass of Ce‐MOFs in the preparation process. In particular, the ZTNs‐Ce20 nanocages display the highest H_2_ evolution rate of 7.46 mmol g^−1^ h^–1^, which is higher than most reported works (Table S3, Supporting Information) and ≈3 times higher than that of pristine ZIS. However, with a further increase in the addition of Ce‐MOFs, the photocatalytic activities of ZTNs‐Cex (*x* = 30, 40, 60) decreases; notably, they are still higher than that of pristine ZIS. Based on the photocatalysis results, the optimal doping amount of Ce in ZTNs‐Cex can be further confirmed, suggesting that ZTNs‐Ce20 possess a suitable bandgap and position for photocatalytic H_2_ evolution. More importantly, the outstanding photocatalytic performance of ZTNs‐Ce20 also demonstrates the superiority of the hollow nanocage structure. The H_2_‐forming rate of ZTNs‐Ce20 increases over 4 h period, while that of pristine ZIS clearly decreases after reacting for 3 h (Figure 6b). These results can be ascribed to the unique hierarchical hollow cage structure and Ce doping, which are beneficial to the efficient separation/transfer of photoexcited charge carriers. The apparent quantum efficiencies (AQEs) of ZTNs‐Ce20 under various monochromatic light irradiation were detected, and the results are shown in Figure 6c. The AQE of ZTNs‐Ce20 reaches a value as high as 6.56% at 380 nm (Table S4, Supporting Information). Notably, the AQE of ZTNs‐Ce20 is greatly affected by the wavelength of incident light. The AQE decreases with an increasing wavelength of incident light, which matches well with the optical absorption spectrum. These results indicate that the strong light‐absorption capacity of the material can generate many more charge carriers, thus resulting in higher photocatalytic activity. To investigate the stability of ZTNs‐Ce20 in the photocatalytic hydrogen production reaction, ZTNs‐Ce20 photocatalysts were used to conduct this reaction for 16 h. No apparent deactivation is observed in four 4 h cycles (Figure 6d). These results reveal that the ZTNs‐Ce20 photocatalysts display high stability similar to most of the reported catalysts (Table S3, Supporting Information). The stability of the ZTNs‐Ce20 photocatalysts is also proven by the XRD results of the sample before and after the photocatalytic reaction. The XRD pattern comparison (Figure S10, Supporting Information) of the samples before and after reaction reveal its unchanged crystal structure.

### 2.3 Charge‐Separation and Proposed Mechanism

**Figure 7:**
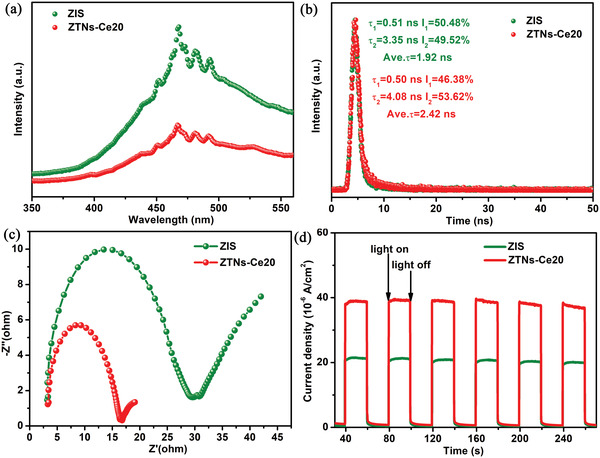
a) Room‐temperature PL spectra, b) the time‐resolved PL (TRPL) spectra, c) EIS spectra, and d) transient photocurrent response of ZIS and ZTNs‐Ce20.

Photoluminescence (PL) spectroscopy was used to test the efficiency of photogenerated carrier separation. As shown in **Figure** **7**a, ZTNs‐Ce20 shows a much weaker PL peak intensity than pristine ZIS, suggesting restrained photoinduced electron–hole pair recombination and indicating that more electrons can participate in the photocatalytic reaction. To further probe the charge carrier dynamics of ZTNs‐Ce20, time‐resolved PL (TRPL) spectroscopy was performed, and the emission decay curves of the samples were fitted by a biexponential kinetics function. As illustrated in Figure 7b, ZTNs‐Ce20 displays a longer average lifetime (2.42 ns) than pristine ZIS (1.92 ns). The obvious increase in the PL lifetime of ZTNs‐Ce20 demonstrates that it can effectively promote the separation of photoinduced electrons and holes.^[^^35]^ In addition, from the electrochemical impedance spectroscopy (EIS) results, we can observe that the ZTNs‐Ce20 photocatalysts have smaller semicircles than pristine ZIS (Figure 7c); thus, the charge transfer resistance at the ZTNs‐Ce20 electrolyte interface is much smaller, suggesting faster interfacial charge transport and more effective separation of photogenerated charge carriers.^[^ ^44^, ^45^ ^]^ As shown in Figure 7d, the photocurrent of ZTNs‐Ce20 is much higher than that of ZIS, implying that the ZTNs‐Ce20 nanocages enhance charge transfer at the interface while suppressing the recombination rate of photogenerated electrons and holes.^[^ ^10^, ^46^ ^]^

The light‐harvesting capability of ZIS and ZTNs‐Cex (*x* = 10, 15, 20, 30, 40, 60) was examined by UV–vis diffuse reflectance spectra (UV–vis DRS). As shown in Figure S11a (Supporting Information), ZIS shows a visible absorption edge at ≈480 nm, and after Ce doping, the absorption edge shows a significant redshift, indicating that Ce has been doped into the crystal structure of ZIS. It is well known that elemental doping will strongly affect the band structure of semiconductors.^[^ ^23^ ^]^ The bandgap of ZTNs‐Ce20 is calculated to be ≈2.68 eV based on the UV–vis DRS results, which is slightly smaller than that of pristine ZIS (2.77 eV) (Figure S11b, Supporting Information). Therefore, the narrowed bandgap of ZTNs‐Ce20 can improve light harvesting.

**Figure 8:**
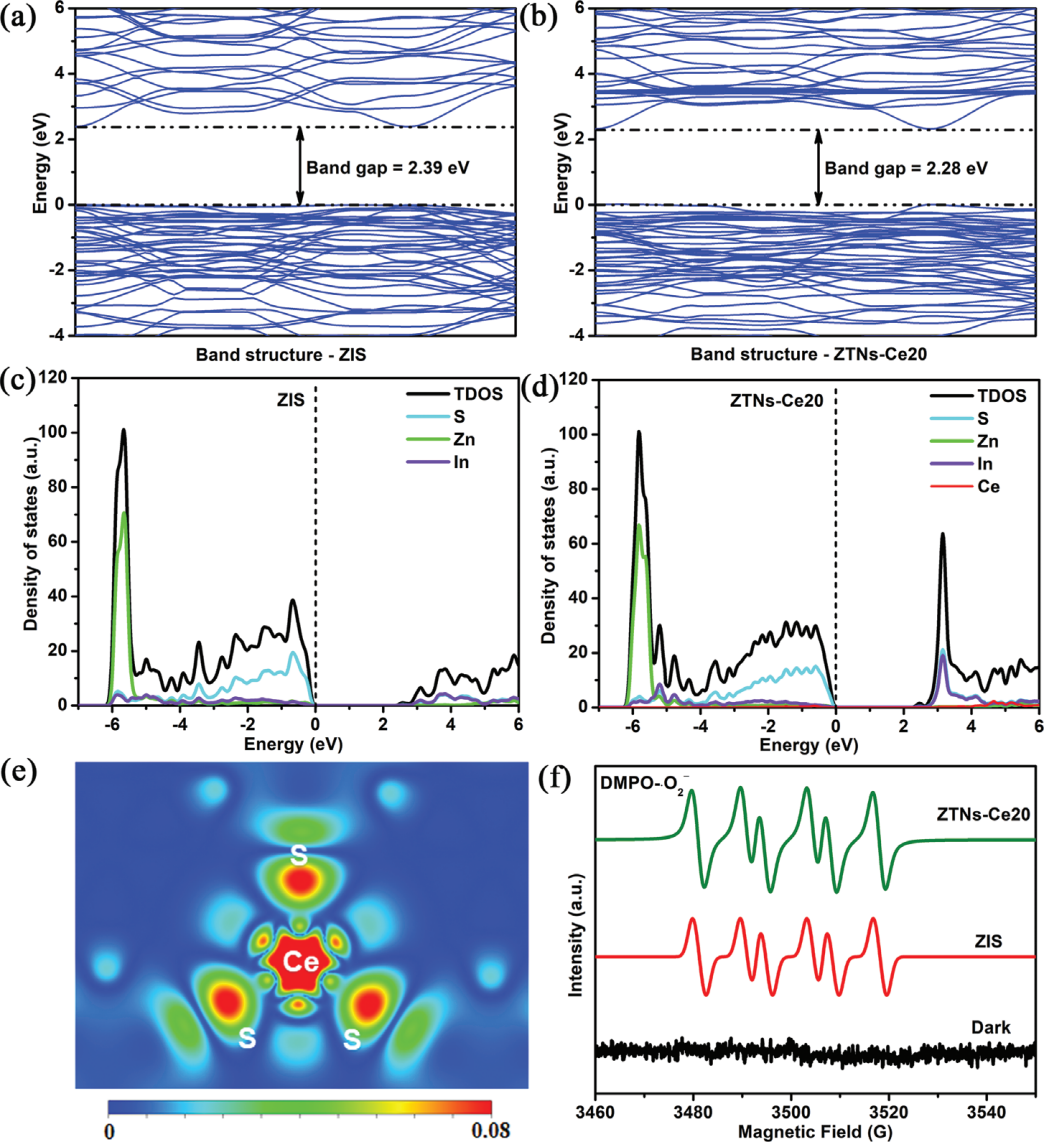
Calculated energy band structures of a) pristine ZIS and b) ZTNs‐Ce20, calculated density of states of c) pristine ZIS and d) ZTNs‐Ce20, e) The electron difference density analysis of ZTNs‐Ce20, and f) DMPO spin‐trapping ESR spectra of ⋅O_2_^−^ over pure ZIS and ZTNs‐Ce20 photocatalysts in the dark and after 10 min of visible light irradiation.

Furthermore, Density functional theory (DFT) calculation models of pristine ZIS and ZTNs‐Ce20 were constructed to acquire deep insight into the effect of Ce doping on the electronic structure and photocatalytic activity. As shown in **Figure** **8**a, the calculated bandgap for pristine ZIS is 2.39 eV, which is much smaller than the experimental value (2.77 eV). This phenomenon of a smaller bandgap calculated by DFT always exists owing to the well‐known limitation of predicting accurate exchange‐correlation potentials.^[^ ^47^ ^]^ After Ce doping, the bandgap of ZTNs‐Ce20 decreases to 2.28 eV. It is clear that Ce doping increases the valence band maximum and narrows the bandgap (Figure 8b). Thus, it can be inferred that the absorption edge of ZTNs‐Ce20 redshifts to longer wavelengths, which is consistent with the UV–vis DRS results. Nevertheless, the hydrogen evolution rate has little contribution to the total hydrogen production under long‐wavelength monochromatic light (Table S3, Supporting Information). Therefore, the ZTNs‐Ce20 photocatalyst has a better hydrogen evolution rate, which is mainly due to its enhanced charge separation and transfer. As observed from the density of states in Figure 8c,d, cerium doping also causes some states to pass through the Fermi level, where they act as electron accepters. These unfilled receptor states at the top of the VB induce the metallic conductive properties of ZTNS‐Ce20, and inhibit the undesirable photoinduced recombination of electrons and holes. Furthermore, the electron difference density analysis of ZTNs‐Ce20 shows that the Ce 3d states at the top of the valence band are delocalized (Figure 8e), which is conducive to the separation and migration of charges and thus increases photocatalytic hydrogen production activity.^[^ ^48^ ^]^

The electron spin‐resonance (ESR) spectroscopy with 5,5‐dimethyl‐1‐pyrroline N‐oxide (DMPO) was employed to detect the photocatalytic reactive species of ⋅O_2_^−^ generated in this reaction system. As seen from Figure 8f, no ESR signal can be observed in the dark, while sextet ESR signals for DMPO‐⋅O_2_^−^ are noticed for pure ZIS and ZTNs‐Ce20 after 10 min of visible light irradiation. The higher DMPO‐⋅O_2_^−^ signal intensities of ZTNs‐Ce20 suggest that the introduction of Ce doping is beneficial to enhancing superoxide anion (·O_2_^−^) generation under visible light irradiation. It also indicates that ZTNs‐Ce20 has a more negative conduction band position than pristine ZIS, that is, a stronger reducing ability.

**Figure 9:**
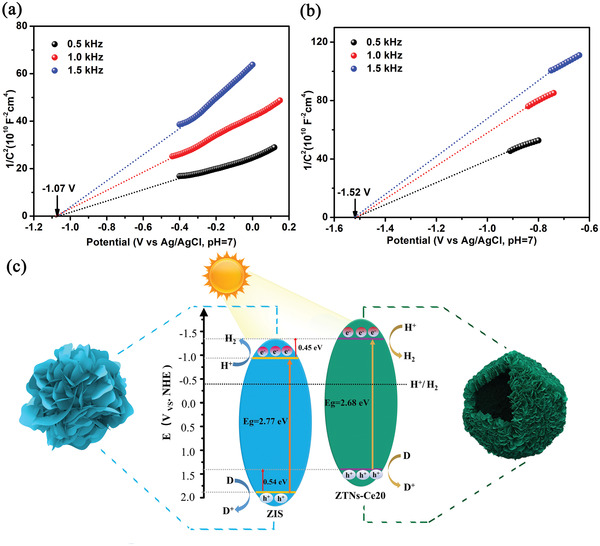
Mott–Schottky plots of ZIS a), ZTNs‐Ce20 b) and schematic illustration of the band structure of the pristine ZIS and ZTNs‐Ce20 samples c).

Consequently, the excellent photocatalytic performance of ZTNs‐Ce20 is related to the photoexcited carrier separation combined with the above discussion. In fact, this is the essential factor that regulates the band structure due to the doping of Ce in ZTNs. Mott–Schottky (M–S) plots of ZIS and ZTNs‐Ce20 were collected at AC frequency of 1.5, 1.0, and 0.5 kHz. As shown in **Figure** **9**a,b, the flat‐band potentials of ZIS and ZTNs‐Ce20 are −1.07 and −1.52 V versus Ag/AgCl (−0.87 and −1.32 V vs NHE, normal hydrogen electrode), respectively. Additionally, according to the bandgap, the Valence‐band (VB) potentials of pristine ZIS and ZTNs‐Ce20 are calculated to be 1.90 and 1.36 eV, respectively. Figure 9c shows a schematic diagram of the possible photocatalytic mechanism. Clearly, the VB of ZTNs‐Ce20 shifts up to ≈0.54 eV compared with that of pristine ZIS. Moreover, the CB of ZTNs‐Ce20 shifts up to ≈0.45 eV, which is consistent with the ESR spectroscopy results. The above results reveal that ZTNs‐Ce20 possesses a much higher mobility and better consumption of photoexcited holes compared with pure ZIS.^[^ ^49^ ^]^ The increase in the CB position allows the photoexcited electrons in ZTNs‐Ce20 much more easily reduce hydrogen ions and generate H_2_ than pure ZIS during photocatalytic hydrogen production.^[^ ^23^ ^]^

In summary, the significant improvements in the catalytic activity of ZTNs‐Ce20 can be ascribed to its hollow hierarchical structure and the regulation of its band structure due to the doping of Ce in the ZTNs; thus, the photocatalyst is endowed with high light‐harvesting and effective charge carrier separation abilities.

## 3 Conclusion

We present novel Ce‐doped ZnIn_2_S_4_ tetrakaidecahedron hollow nanocages with ultrathin nanosheet subunits via a facile one‐pot solvothermal method by using tetrakaidecahedron Ce‐MOF as a template. Notably, the doping of Ce and the formation of ZnIn_2_S_4_ tetrakaidecahedron hollow nanocages with ultrathin nanosheet subunits are simultaneously induced by the Ce‐MOFs, making this groundbreaking work. As a photocatalyst, these tetrakaidecahedron hollow hierarchical nanocages ensure a larger surface area, more active site exposure and adequate light harvesting. As a result, optimal Ce‐doped ZnIn_2_S_4_ tetrakaidecahedron nanocages, namely, ZTNs‐Ce20, exhibit a high photocatalytic activity for hydrogen evolution (7.46 mmol h^−1^ g^−1^) and a high AQE of 6.56% at 380 nm, which is 3 times higher than that of pristine ZIS (2.61 mmol h^−1^ g^−1^). This work highlights the strategy of using MOFs as both a template and dopant to construct nonspherical hollow nanocage photocatalysts with elemental doping, which will pave the way for the design of new and efficient photocatalysts.

## 4 Experimental Section

### Materials

Cerium (IV) ammonium nitrate ((NH_4_)_2_Ce(NO_3_)_6_), 1,4‐dicarboxybenzene, sodium perchlorate monohydrate (NaClO_4_·H₂O), F127 (PEO_106_PPO_70_PEO_106_), glycerol, zinc chloride (ZnCl_2_), indium chloride tetrahydrate (InCl_3_·4H_2_O), and TAA, were purchased from Aladdin Bio‐Chem Technology Co., Ltd. Triethanolamine (TEOA), glycerol, HCl (35%) were obtained from Sinopharm Chemical Reagent Co. Ltd. (China). All chemicals were analytical purity and used without further treatment. Deionized water was utilized in the entire experiment.

### Synthesis of Ordered Large‐Pore Tetrakaidecahedron Ce‐MOFs

Uniform ordered large‐pore tetrakaidecahedron Ce‐MOFs particles are synthesized by adapted from known literature procedure.^[^ ^50^ ^]^ Acetic acid (0.3 mL, 5.1 mmol) and NaClO_4_·H₂O (500 mg, 3.5 mmol) were added into the solution with 100 mg F127 and 6 mL deionized water. Then, a homogeneous solution was obtained by stirring. Subsequently, (NH_4_)_2_Ce(NO_3_)_6_ (548 mg, 1 mmol) and 1,4‐dicarboxybenzene (166 mg, 1 mmol) were added and stirred at 60 °C for 20 min. The as‐synthesized samples were collected by centrifugation, washed with distilled water and *N,N*‐dimethylformamide. To remove the template, the resultant solids were soaked in ethanol for two days at 60 °C, during which time the ethanol was changed every day. The final products were dried overnight at 60 °C under vacuum.

### Synthesis of ZIS Tetrakaidecahedron Nanocages (ZTNs‐Cex)

Different amounts of Ce‐MOFs (10, 15, 20, 30, 40, 60 mg) were dissolved into 8 mL of water (pH at 2.5), 2 mL of glycerol, and stirred for 30 min, and followed by the addition of InCl_3_·4H_2_O (117.2 mg), ZnCl_2_ (27.2 mg), and TAA (60.1 mg). Subsequently, after stirred for 10 min, the obtained mixture was put into the 80 °C oil bath lately for 2 h. Then, the obtained samples were gathered, washed with ethanol, and then dried at 60 °C in vacuum. The corresponding composites were labeled as ZTNs‐Cex (the suffix x indicates the addition mass of the Ce‐MOF).

### Material Characterizations

The data of powder XRD of the samples were measured on Bruker D8 advance X‐ray diffractometer with Cu K*α* radiation in a scanning range of 10°–80° to characterize the crystal phases. FESEM (Zeiss Sigma‐500), TEM (JEOL JEM‐2100F), and elemental mapping images were recorded to examine the morphology and structure of the samples. Determination of the surface chemical states was undertaken by the XPS (EscaLab Xi+) with the reference of C1s peak (284.6 eV). The N_2_ adsorption–desorption isotherms were carried out on a Quantachrome surface area analyzer. The specific surface areas were obtained by the BET method, while the pore size distributions were identified from the adsorption branch of the isotherm. The UV–vis DRS and PL spectra were measured using Hitachi UH4150 UV–visible spectrophotometer and Edinburgh Analytical Instruments FLS980. Labsolar‐IIIAG photocatalytic system (Beijing Perfect light Co., Ltd.) was used under the irradiation of 300 W xenon lamp (PLS‐SXE300) with a UV‐CUT filter (*λ* ≥ 400 nm).

### Photocatalytic Activity Test

Typically, 5 mg of photocatalyst was dispersed to aqueous solution (30 mL) containing 3 mL triethanolamine as sacrificial reagents. A 300 W Xe lamp (PLS‐SXE300) with a 400 nm cutoff filter was utilized as the light source, and the photocatalytic experiments were carried out in an online Labsolar‐IIIAG photocatalytic system (Beijing Perfect light Co., Ltd.). Prior the H_2_ evolution experiment, the reactor was thoroughly degassed for 30 min to remove air. The temperature was controlled at 25 °C by cooling circulating water during the reaction process. The gas chromatography (Shanghai Kechuang, GC910) was used to detect the generated H_2_. The AQEs for photocatalytic hydrogen production were measured by means of placing different monochromatic light filters (380, 400, 420, 450 nm), and the power meter (Thorlabs PM100D) was performed to measure the number of incident photons. The AQE was calculated by equation

(1) $$\mathrm{AQE}\;=\frac{2\times \mathrm{the}\;\mathrm{number}\;\mathrm{of}\;\mathrm{evolved}\;H_{2}\;\mathrm{molecules}}{\mathrm{the}\;\mathrm{number}\;\mathrm{of}\;\mathrm{incident}\;\mathrm{photons}}\;\times 100\%$$

### Photoelectrochemical Study

A standard three‐electrode cell on a CHI 660E was used to measure the photoelectrochemical properties, in which Ag/AgCl electrode, Pt plate, and photocatalyst film on fluorinated tin oxide (FTO) glass acted as the reference electrode, counter electrode, and working electrode, respectively, with Na_2_SO_4_ (0.5 mol L^−1^) aqueous solution as an electrolyte. The work electrode was prepared through a spray‐coating method, with a paste containing 3 mg ethyl cellulose, 30 mg catalyst, and 5 mL EtOH rolled by a glass rod on the FTO glass, and then dried at 120 °C for 2 h. In the photocurrent measurements, the light source was a 300 W Xe lamp with a 400 nm cutoff filter. The EIS was acquired in the frequency range from 10^5^ to 0.1 Hz.

### Computational Methods

DFT calculations were undertook using the Vienna ab initio Simulation Program (VASP).^[^ ^51^, ^52^ ^]^ In the calculation process, the generalized gradient approximation (GGA) in the Perdew–Burke–Ernzerhof (PBE) take place and a cutoff energy of 520 eV for plane wave basis set was adopted.^[^ ^53^ ^]^ A 7×7×1 Monkhorst‐Pack grid was used for sampling the Brillouin zones at structure optimization.^[^ ^54^ ^]^ The projector augmented wave (PAW) method was chosen to depict the ion‐electron interactions.^[^ ^55^ ^]^ The convergence criteria of structure optimization was the maximum force on each atom less than 0.01 eV Å^−1^ with an energy change less than 1 × 10^−6^ eV.

## Conflict of Interest

The authors declare no conflict of interest.

## Supporting information

Supporting Information

## Data Availability

Research data are not shared.

## References

[1] X. X. Zhao , J. R. Feng , J. W. Liu , J. Lu , W. Shi , G. M. Yang , G. C. Wang , P. Y. Feng , P. Cheng , Adv. Sci. 2018, 5, 1700590. 10.1002/advs.201700590 PMC5908348 29721410

[2] Y. Shi , A. F. Yang , C. S. Cao , B. Zhao , Coord. Chem. Rev. 2019, 390, 50.

[3] M. Y. Xing , B. C. Qiu , M. M. Du , Q. H. Zhu , L. Z. Wang , J. L. Zhang , Adv. Funct. Mater. 2017, 27, 1702624.

[4] C. Cai , Y. Teng , J. H. Wu , J. Y. Li , H. Y. Chen , J. H. Chen , D. B. Kuang , Adv. Funct. Mater. 2020, 30, 2001478.

[5] M. Sachs , R. S. Sprick , D. Pearce , S. A. J. Hillman , A. Monti , A. A. Y. Guilbert , N. J. Brownbill , S. Dimitrov , X. Y. Shi , F. Blanc , M. A. Zwijnenburg , J. Nelson , J. R. Durrant , A. I. Cooper , Nat. Commun. 2018, 9, 4968. 30470759 10.1038/s41467-018-07420-6 PMC6251929

[6] I. Vamvasakis , B. Liu , G. S. Armatas , Adv. Funct. Mater. 2016, 26, 8062.

[7] L. Ma , K. Chen , F. Nan , J. H. Wang , D. J. Yang , L. Zhou , Q. Q. Wang , Adv. Funct. Mater. 2016, 26, 6076.

[8] B. Cao , G. S. Li , H. X. Li , Appl. Catal., B 2016, 194, 42.

[9] J. G. Wang , P. Zhang , X. Li , J. Zhu , H. X. Li , Appl. Catal., B 2013, 134–135, 198.

[10] L. Tian , S. X. Min , F. Wang , Appl. Catal., B 2019, 259, 118029.

[11] S. N. Xiao , W. R. Dai , X. Y. Liu , D. L. Pan , H. J. Zou , G. S. Li , G. Q. Zhang , C. L. Su , D. Q. Zhang , W. Chen , H. X. Li , Adv. Energy Mater. 2019, 9, 1900775.

[12] P. Zhang , D. Y. Luan , X. W. Lou , Adv. Mater. 2020, 32, 2004561. 10.1002/adma.202004561 32815207

[13] Y. Goto , T. Hisatomi , Q. Wang , T. Higashi , K. Ishikiriyama , T. Maeda , Y. Sakata , S. Okunaka , H. Tokudome , M. Katayama , S. Akiyama , H. Nishiyama , Y. Inoue , T. Takewaki , T. Setoyama , T. Minegishi , T. Takata , T. Yamada , K. Domen , Joule 2018, 2, 509.

[14] P. Zhang , L. Yu , X. W. Lou , Angew. Chem., Int. Ed. 2018, 57, 15076. 10.1002/anie.201808104 30247798

[15] H. H. Ou , L. H. Lin , Y. Zheng , P. J. Yang , Y. H. Fang , X. C. Wang , Adv. Mater. 2017, 29, 1700008. 10.1002/adma.201700008 28401588

[16] J. Zhang , J. G. Yu , Y. M. Zhang , Q. Li , J. R. Gong , Nano Lett. 2011, 11, 4774. 21981013 10.1021/nl202587b

[17] X. X. Zhao , J. R. Feng , J. Liu , W. Shi , G. M. Yang , G. C. Wang , P. Cheng , Angew. Chem., Int. Ed. 2018, 57, 9790. 10.1002/anie.201805425 29888442

[18] I. Tsuji , H. Kato , A. Kudo , Angew. Chem., Int. Ed. 2005, 44, 3565. 10.1002/anie.200500314 15880535

[19] A. Iwase , S. Yoshino , T. Takayama , Y. H. Ng , R. Amal , A. Kudo , J. Am. Chem. Soc. 2016, 138, 10260. 27459021 10.1021/jacs.6b05304

[20] X. C. Wang , K. Maeda , X. F. Chen , K. Takanabe , K. Domen , Y. D. Hou , X. Z. Fu , M. Antonietti , J. Am. Chem. Soc. 2009, 131, 1680. 19191697 10.1021/ja809307s

[21] T. Zhu , H. B. Wu , Y. B. Wang , R. Xu , X. W. Lou , Adv. Energy Mater. 2012, 2, 1497.

[22] B. Xu , P. L. He , H. L. Liu , P. P. Wang , G. Zhou , X. Wang , Angew. Chem., Int. Ed. 2014, 53, 2339. 10.1002/anie.201310513 24478173

[23] W. L. Yang , L. Zhang , J. F. Xie , X. D. Zhang , Q. H. Liu , T. Yao , S. Q. Wei , Q. Zhang , Y. Xie , Angew. Chem., Int. Ed. 2016, 55, 6716. 10.1002/anie.201602543 27100950

[24] Y. J. Chen , G. H. Tian , Z. Y. Ren , K. Pan , Y. H. Shi , J. Q. Wang , H. G. Fu , ACS Appl. Mater. Interfaces 2014, 6, 13841. 25057818 10.1021/am5032083

[25] X. Y. Dang , M. S. Xie , F. F. Dai , J. N. Guo , J. Liu , X. Q. Lu , J. Mater. Chem. A 2021, 9, 14888.

[26] G. C. Zuo , Y. T. Wang , W. L. Teo , A. M. Xie , Y. Guo , Y. X. Dai , W. Q. Zhou , D. Jana , Q. M. Xian , W. Dong , Y. L. Zhao , Angew. Chem., Int. Ed. 2020, 59, 11287. 10.1002/anie.202002136 32250502

[27] G. C. Zuo , Y. T. Wang , W. L. Teo , Q. M. Xian , Y. L. Zhao , Appl. Catal., B 2021, 291, 120126.

[28] C. Du , B. Yan , G. W. Yang , Nano Energy 2020, 76, 105031.

[29] W. S. Jiang , Y. J. Zhao , X. P. Zong , H. D. Nie , L. J. Niu , L. An , D. Qu , X. Y. Wang , Z. H. Kang , Z. C. Sun , Angew. Chem., Int. Ed. 2021, 60, 6124. 10.1002/anie.202015779 33471365

[30] Y. Kageshima , T. Kawanishi , D. Saeki , K. Teshima , K. Domen , H. Nishikiori , Angew. Chem., Int. Ed. 2021, 60, 3654. 10.1002/anie.202011705 33166019

[31] J. M. Qian , T. T. Wang , Z. M. Zhang , Y. G. Liu , J. F. Li , D. Q. Gao , Nano Energy 2020, 74, 104948.

[32] Z. Zhu , C. C. Ma , K. S. Yu , Z. Y. Lu , Z. Liu , P. W. Huo , T. Xu , Y. S. Yan , Appl. Catal., B 2020, 268, 118432.

[33] J. Wu , P. Huang , H. T. Fan , G. Wang , W. S. Liu , ACS Appl. Mater. Interfaces 2020, 12, 30304. 32543170 10.1021/acsami.0c03929

[34] Z. Shayegan , F. Haghighat , C. S. Lee , Chem. Eng. J. 2020, 401, 125932.

[35] S. B. Wang , B. Y. Guan , X. Wang , X. W. Lou , J. Am. Chem. Soc. 2018, 140, 15145. 30380851 10.1021/jacs.8b07721

[36] J. H. Sun , J. S. Zhang , M. W. Zhang , M. Antonietti , X. Z. Fu , X. C. Wang , Nat. Commun. 2012, 3, 1139.

[37] S. B. Wang , B. Y. Guan , Y. Lu , X. W. Lou , J. Am. Chem. Soc. 2017, 139, 17305. 29116762 10.1021/jacs.7b10733

[38] B. C. Qiu , Q. H. Zhu , M. M. Du , L. G. Fan , M. Y. Xing , J. L. Zhang , Angew. Chem., Int. Ed. 2017, 56, 2684. 10.1002/anie.201612551 28141900

[39] W. X. Liu , J. J. Huang , Q. Yang , S. J. Wang , X. M. Sun , W. N. Zhang , J. F. Liu , F. W. Huo , Angew. Chem., Int. Ed. 2017, 56, 5512. 10.1002/anie.201701604 28334498

[40] T. T. Hou , N. C. Luo , Y. T. Cui , J. M. Lu , L. Li , K. E. MacArthure , M. Heggene , R. T. Chen , F. T. Fan , W. M. Tian , S. Y. Jin , F. Wang , Appl. Catal., B 2019, 245, 262.

[41] C. C. Li , X. H. Liu , G. Z. Lu , Y. Q. Wang , Chin. J. Catal. 2014, 35, 1364.

[42] F. L. Zhang , X. Zhang , Z. P. Hao , G. X. Jiang , H. L. Yang , S. Q. Qu , J. Hazard. Mater. 2018, 342, 749. 28918293 10.1016/j.jhazmat.2017.09.014

[43] H. Y. Liu , S. Ai , Y. J. Liu , H. M. Zeng , H. M. Da , Y. L. Liu , Y. Q. Chai , R. Yuan , Chem. Commun. 2020, 56, 14275. 10.1039/d0cc06111e 33125007

[44] Y. R. Zheng , J. Dong , C. P. Huang , L. G. Xia , Q. Wu , W. F. Yao , Q. J. Xu , Appl. Catal., B 2020, 260, 118220.

[45] Z. X. Lin , C. T. Shao , S. J. Jiang , C. Z. Sun , S. Q. Song , Appl. Catal., B 2020, 268, 118742.

[46] W. C. Wang , X. Q. Bai , Q. Ci , L. L. Du , X. G. Ren , D. L. Phillips , Adv. Funct. Mater. 2021, 31, 2103978.

[47] M. Dong , P. Zhou , C. J. Jiang , B. Cheng , J. G. Yu , Chem. Phys. Lett. 2017, 668, 1.

[48] P. F. Wang , Z. R. Shen , Y. G. Xia , H. T. Wang , L. R. Zheng , W. Xi , S. H. Zhan , Adv. Funct. Mater. 2019, 29, 1807013.

[49] C. Du , B. Yan , Z. Y. Lin , G. W. Yang , J. Mater. Chem. A 2020, 8, 207.

[50] K. Li , J. Yang , R. Huang , S. L. Lin , J. L. Gu , Angew. Chem., Int. Ed. 2020, 59, 14124. 10.1002/anie.202006124 32400955

[51] G. Kresse , J. Furthmüller , Comput. Mater. Sci. 1996, 6, 15.

[52] G. Kresse , J. Furthmüller , Phys. Rev. B 1996, 54, 11169. 10.1103/physrevb.54.11169 9984901

[53] J. P. Perdew , K. Burke , M. Ernzerhof , Phys. Rev. Lett. 1996, 77, 3865. 10062328 10.1103/PhysRevLett.77.3865

[54] H. J. Monkhorst , J. D. Pack , Phys. Rev. B 1976, 13, 5188.

[55] P. E. Blöchl , Phys. Rev. B 1994, 50, 17953. 10.1103/physrevb.50.17953 9976227

