# Supplementary material for: One‐Step MOF‐Templated Strategy to Fabrication of Ce‐Doped ZnIn2S4 Tetrakaidecahedron Hollow Nanocages as an Efficient Photocatalyst for Hydrogen Evolution
Source: Adv Sci (Weinh). 2022 Jan 9;9(9):2104579. doi: 10.1002/advs.202104579 (PMC8948573; doi:10.1002/advs.202104579)
Supplement: Supplementary file 1 — Supporting Information [file ADVS-9-2104579-s001.pdf]

ADVANCED SCIENCE
Open Access

Supporting Information

for *Adv. Sci.,* DOI: 10.1002/advs.202104579

One-step MOF-templated strategy to fabrication of Ce-doped ZnIn2S4 tetrakaidecahedron hollow nanocages as an efficient photocatalyst for hydrogen evolution

*Hui-Tao Fan, Yu-Jie Jin, Ke-Cheng Liu, Wei-Sheng Liu**

One-step MOF-templated strategy to fabrication of Ce-doped $ZnIn_2S_4$ tetrakaidecahedron hollow nanocages as an efficient photocatalyst for hydrogen evolution

Hui-Tao Fan[a,b], *Yu-Jie Jin*[b], *Ke-Cheng Liu*[b], *Wei-Sheng Liu**[a]

Dr. H. Fan, Prof. W. Liu

Key Laboratory of Nonferrous Metal Chemistry and Resources Utilization of Gansu Province and State Key Laboratory of Applied Organic Chemistry, College of Chemistry and Chemical Engineering, Lanzhou University,

Lanzhou 730000, P.R. China

E-mail: liuws@lzu.edu.cn

Y. Jin, K. Liu

College of Chemistry and Pharmaceutical Engineering,

Nanyang Normal University, Nanyang 473061, P.R. China

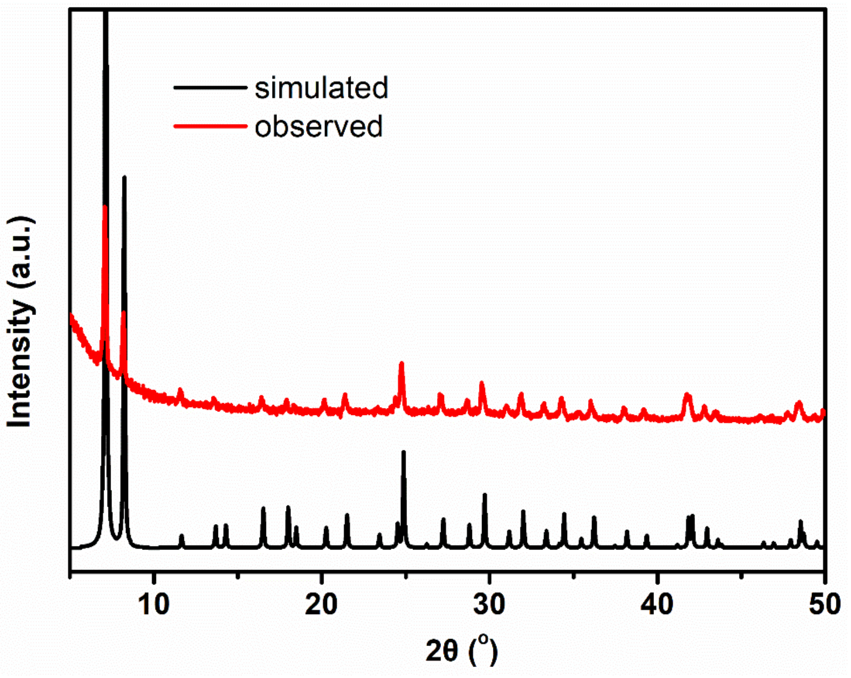

Figure S1. XRD pattern of Ce-MOFs.

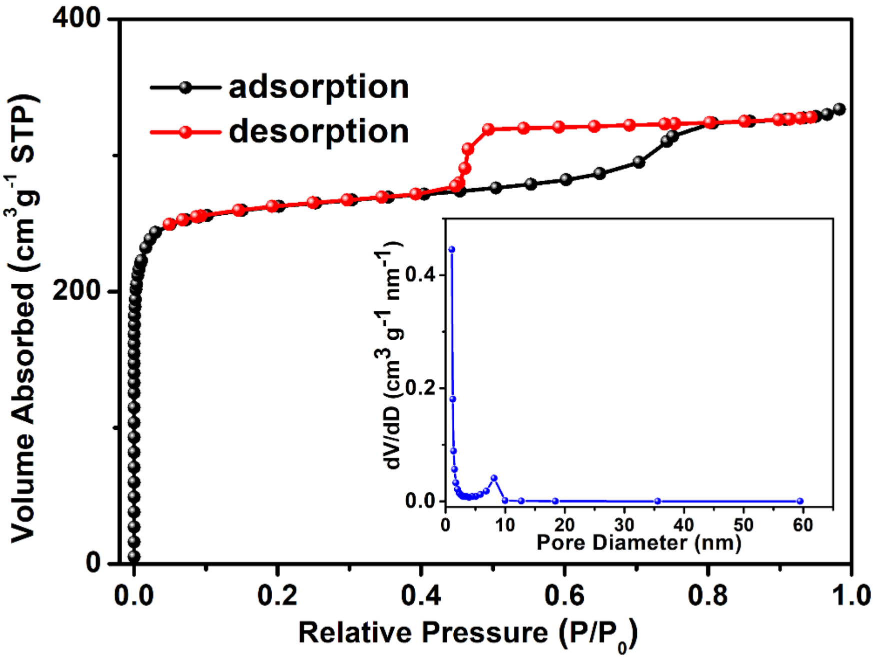

Figure S2. N_2 sorption isotherms and the corresponding pore size distribution of Ce-MOFs.

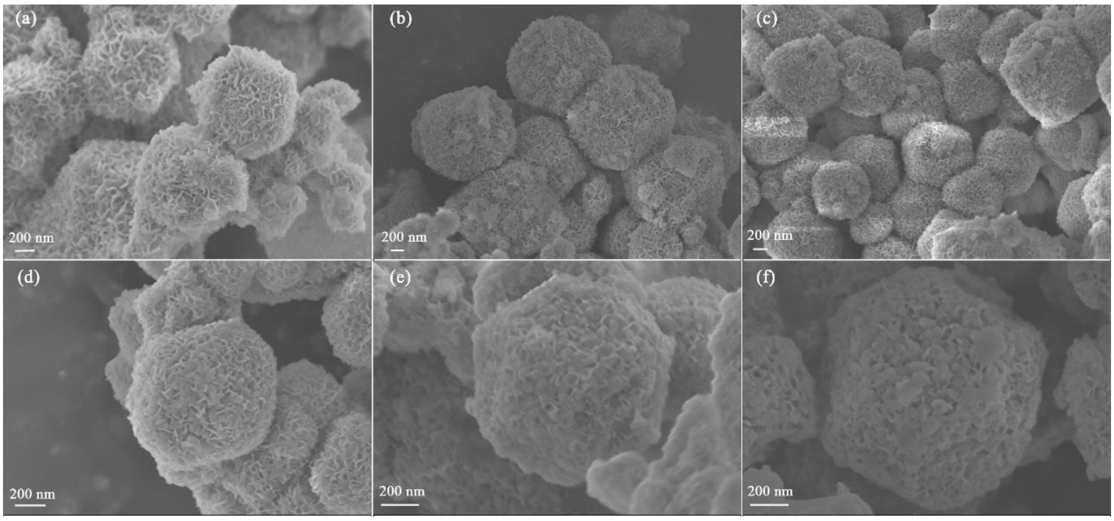

Figure S3. FESEM images of ZTNs-Ce obtained with different content of Ce-MOFs: (a) 10 mg, (b) 15 mg, (c) 20 mg, (d) 30 mg, (e) 40 mg, (f) 60 mg, while other conditions remain unchanged.

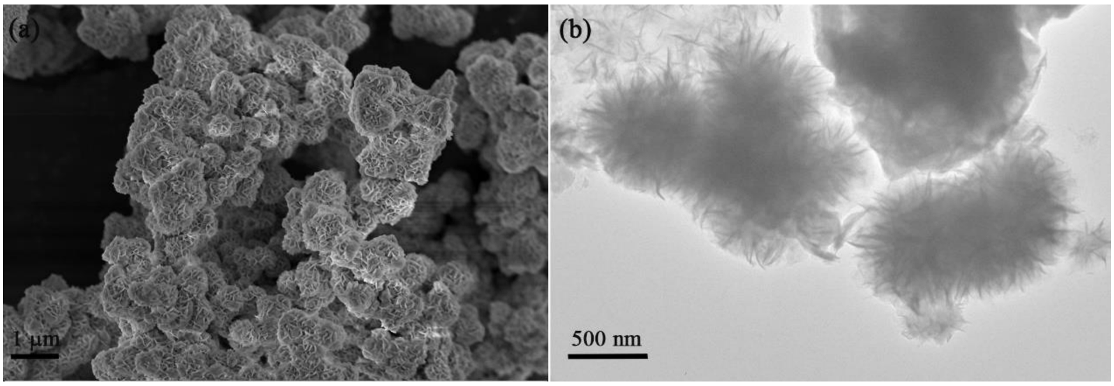

Figure S4. FESEM and TEM image of pristine ZIS.

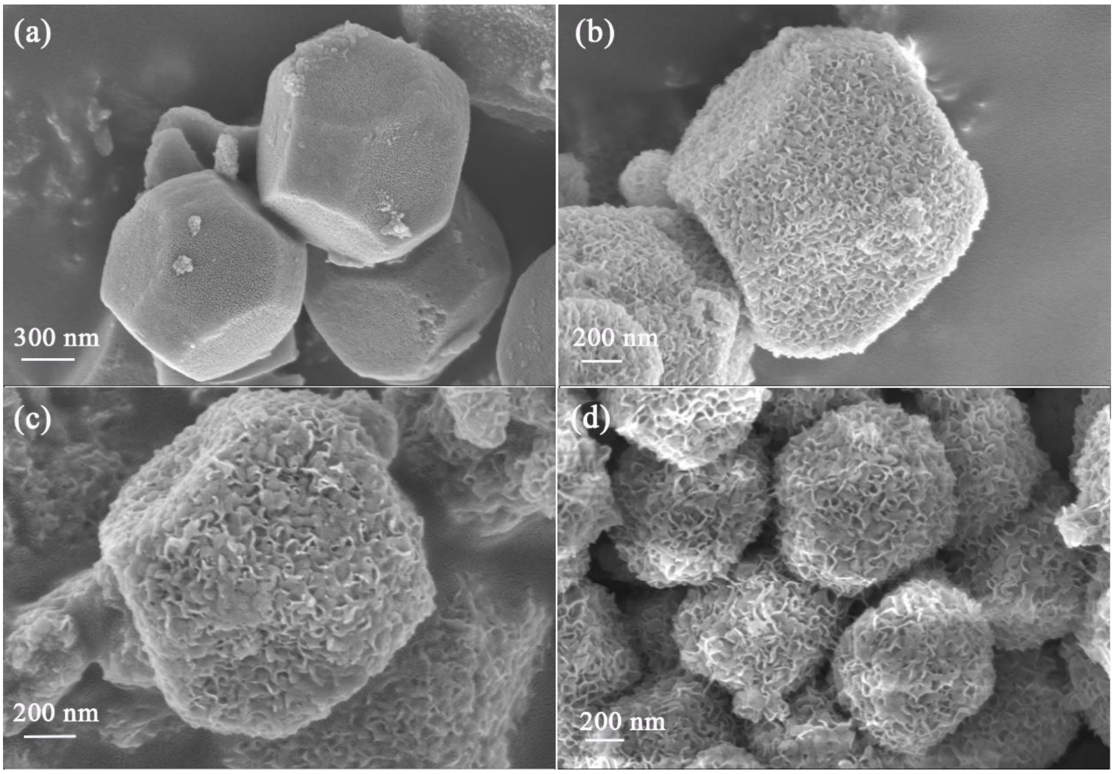

Figure S5. FESEM images of the samples in different reaction stages: (a-d) 10 min, 30 min, 1 h, and 2 h, respectively.

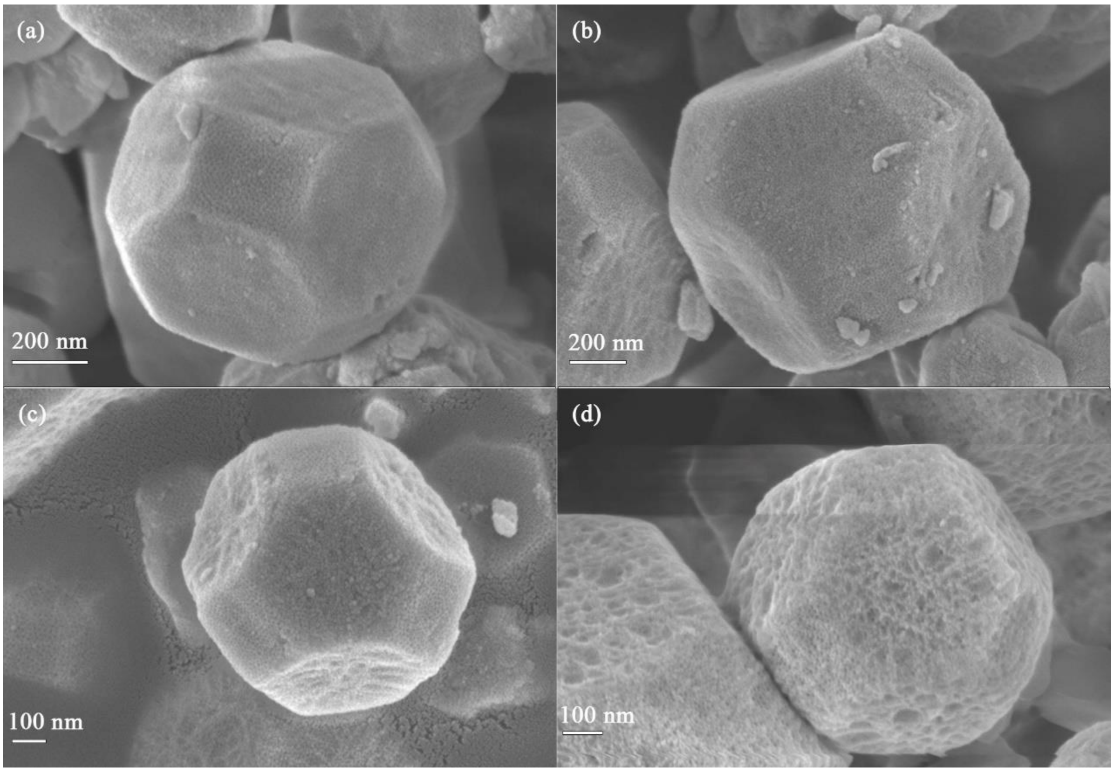

Figure S6. FESEM images of the Ce-MOFs were immersed in HCl solutions stirring

at 80 °C for 2 h: (a) pH = 7, (b) pH = 5, (c) pH = 3, (d) pH = 1.

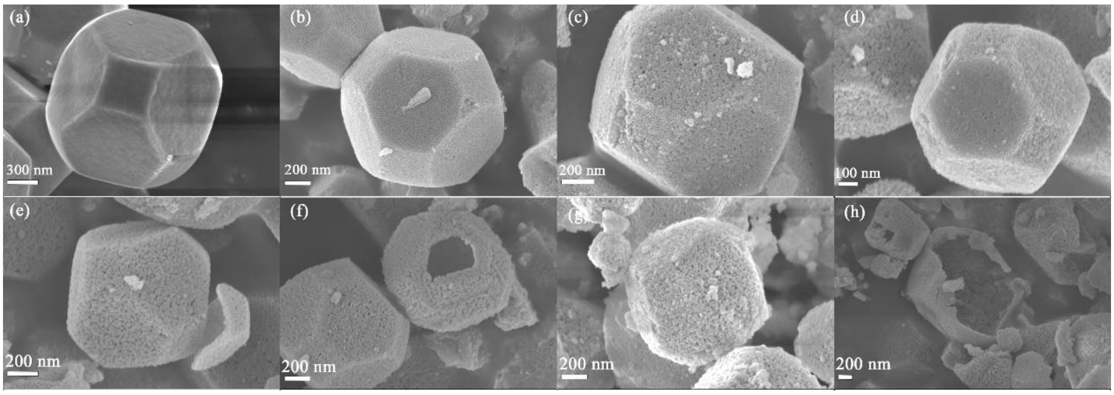

Figure S7. FESEM images of the Ce-MOFs were immersed in TAA solutions (30 mg TAA was dissolved in 20 mL aqueous solution with pH=2.5) stirring at 80 °C for different times: (a) 0 min, (b) 10 min, (c) 15 min, (d) 20 min, (e) 30 min, (f) 40 min, (g) 50 min, (h) 60 min.

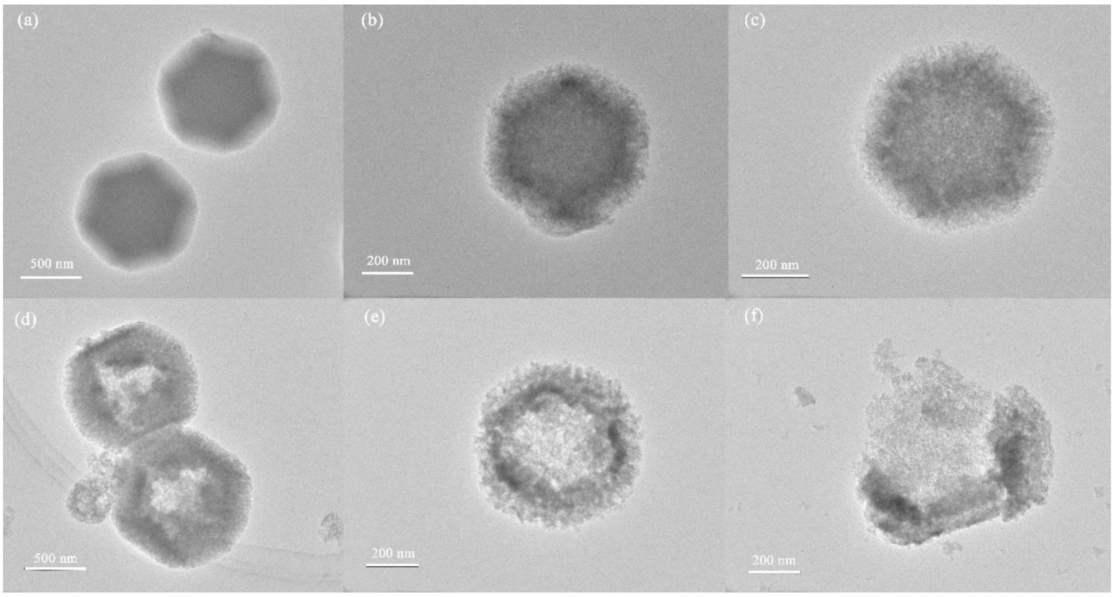

Figure S8. TEM images of the Ce-MOFs were immersed in TAA solutions (30 mg TAA was dissolved in 20 mL aqueous solution with pH=2.5) stirring at 80 °C for different times: (a) 10 min, (b) 20 min, (c) 30 min, (d) 40 min, (e) 50 min, (f) 60 min.

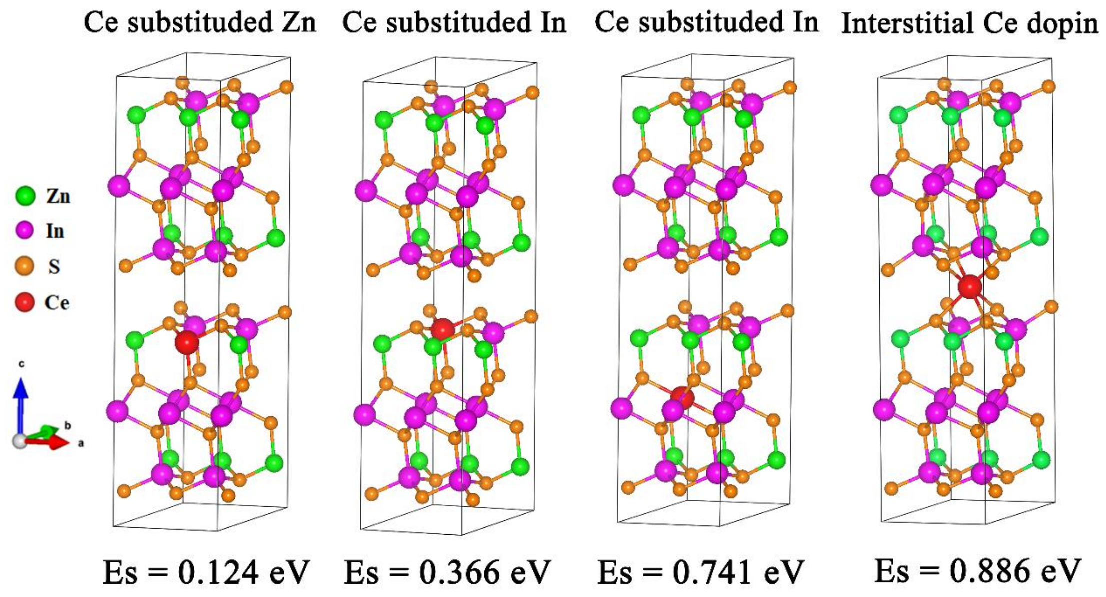

Figure S9. Structure illustration and formation energy of atomic substitutional forms.

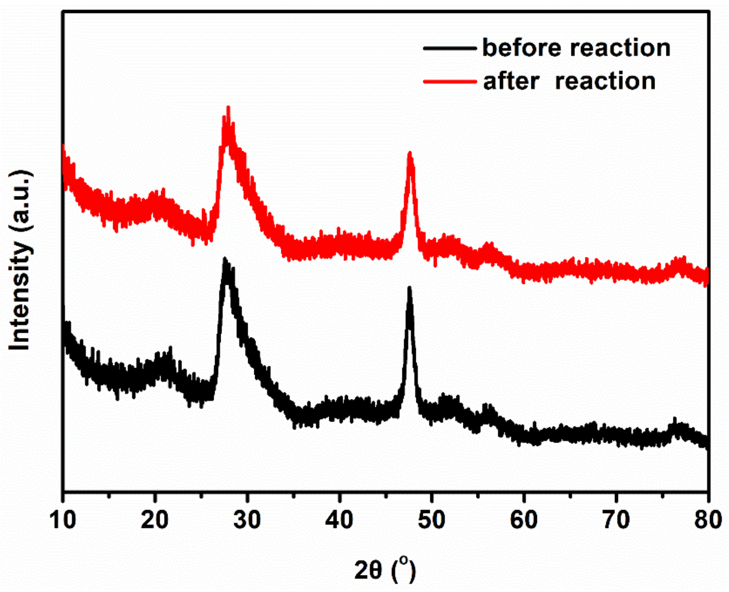

Figure S10. XRD patterns of ZTNs-Ce20 before and after photocatalytic H_2 evolution reactions.

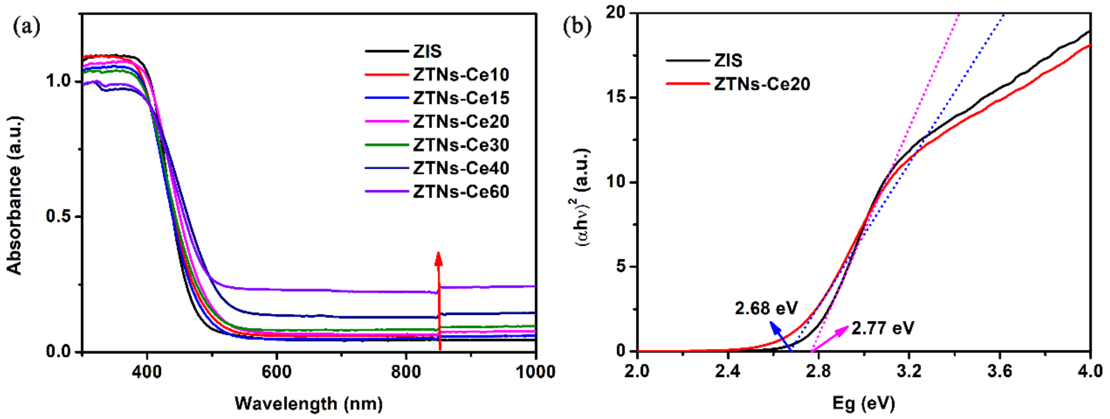

Figure S11. (a) UV-Vis DRS spectra of ZIS and ZTNs-Cex(x=10, 15, 20, 30, 40, 60), (b) Band gap that estimated by a related curve of $(\alpha h\nu)^2$ versus photon energy of ZIS and ZTNs-Ce20.

Table S1. Different Methods for synthesizing $ZnIn_2S_4$-based hollow structure photocatalysts

Catalyst	Synthetic method	Morphology	Temperature/ reaction time	Application	Ref.
ZTNs-Ce20	One step template method	Tetrakaidecahedron hollow nanocages	80 ˚C/2h	Photocatalytic hydrogen production 7.46 mmol·h^{-1}·g^{-1}	This work
$Co_9S_8@ZnIn_2S_4$	Multi-step template method	Polyhedron hollow nanocages	80 ˚C/2h	Photocatalytic hydrogen production 6.25 mmol·h^{-1}·g^{-1}	1
P-ZIS	Multi-step hydrothermal method	Hollow microspheres	180 ˚C/5h	Photocatalytic hydrogen production 0.39 mmol·h^{-1}·g^{-1}	2
TiO_2@ZIS	Multi-step template method	Hollow nanospheres	180 ˚C/3h	Photocatalytic hydrogen production 1.13 mmol·h^{-1}·g^{-1}	3
$CdS/ZnIn_2S_4$	Multi-step template method	Hollow cubes	80 ˚C/2h	Photocatalytic hydrogen production 0.54 mmol·h^{-1}·g^{-1}	4
$NiS@ZnIn_2S_4$	Multi-step template method	Hollow nanospheres	80 ˚C/2h	Photocatalytic hydrogen production 1.24 mmol·h^{-1}·g^{-1}	5

$Ni_{1-x}Co_xSe_2$-C/$ZnIn_2S_4$	Multi-step template method	Hollow cubes	80 °C/2h	Photocatalytic hydrogen production 5.1 mmol·h^{-1}·g^{-1}	6

Table S2. The weight percentage content of elements from ICP-AES and atomic ratio of Zn : In in samples

Samples	Zn (wt%)	In (wt%)	Ce (wt%)	Atomic ratio (Zn:In)
ZTNs-10	12.60	49.35	1.39	1:2.23
ZTNs-15	12.27	48.79	1.52	1:2.26
ZTNs-20	11.22	49.22	1.79	1:2.49
ZTNs-30	9.96	45.50	1.84	1:2.60
ZTNs-40	9.89	47.61	1.95	1:2.72
ZTNs-60	8.47	43.76	1.99	1:2.94

Table S3. Comparison of photocatalytic H_2 generation performance and stability with reported literatures.

Catalyst	Reaction Conditions	Light Source	H_2 evolution rete (mmol $h^{-1}g^{-1}$)	AQE (%)	Cycle-index	Ref.
ZTNs-Ce20	30 mL of aqueous solution containing 3 mL of triethanolamine	300 W xenon lamp with a 400 nm cutoff filter	7.46	6.56 at 380 nm	every 4 h reaction for four cycles	This work
Co_9S_8@$ZnIn_2S_4$	5 ml deionized water and 1 ml triethanolamine	300 W xenon lamp with a 400 nm cutoff filter	6.25	/	every 4 h reaction for five cycles	1
$ZnIn_2S_4$@NH2-MIL-125(Ti)$_4$	100 mL of aqueous solution containing 0.25 M Na_2SO_3 and 0.35 M Na_2S	300 W xenon lamp with a 420 nm cutoff filter	2.20	4.3 at 420 nm	every 4 h reaction for five cycles	7
Ni_2P/$ZnIn_2S_4$	100 mL of aqueous solution	300 W xenon lamp	2.07	7.7 at 420 ±	every 4 h	8

	containing 10 mL of lactic acid	with a 400 nm cutoff filter		20 nm	reaction for five cycles	
$Ti_3C_2T_X$ MXene@$ZnIn_2S_4$	40 mL aqueous solution containing 10 vol% triethanolamine, 3 wt % Pt	300 W xenon lamp with a 420 nm cutoff filter	3.48	11.14 at 420 nm	every 4 h reaction for six cycles	9
RGO/$ZnIn_2S_4$	80 mL of triethanolamine aqueous solution (10 v%), 0.3wt% Pt	LEDs (4×3W 420 nm)	2.64	4.4 at 420 nm	/	10
Co-P/$ZnIn_2S_4$	100 mL of 10 vol% of lactic acid aqueous solution (lactic acid/water).	300 W xenon lamp with a 420 nm cutoff filter	7.84	4.3 at 420 nm	every 3h reaction for five cycles	11
2D/2D g-C_3N_4 nanosheet @ $ZnIn_2S_4$	50 mL of aqueous solution containing 10 mL of triethanolamine	300 W xenon lamp with a 420 nm cutoff filter	2.78	7.05 at 420 nm	every 4 h reaction for four cycles	12
Co/NGC@$ZnIn_2S_4$	5 ml deionized water and 1 ml triethanolamine	300 W xenon lamp with a 400 nm cutoff filte	11.27	5.07 at 420 nm	every 4 h reaction for five cycles	13
Co_9S_8/$ZnIn_2S_4$	90 ml deionized water and 10 ml triethanolamine	300 W Xenon lamp	9.04	4.49 at 420 nm	every 5 h reaction for five cycles	14
RGO/La-$ZnIn_2S_4$	200 mL of deionized water containing 0.25 M Na_2SO_3 and 0.35 M Na_2S, Pt	350 W xenon lamp with a 420 nm cutoff filter	2.26	29.45 at 420 nm	every 3 h reaction for five cycles	15
2D/2D WO_3/$ZnIn_2S_4$	100 mL of aqueous solution containing 0.25 M Na_2SO_3 and 0.35 M Na_2S	300 W xenon lamp with a 420 nm cutoff filter	2.20	/	every 5 h reaction for four cycles	16
MoS_2QDs@Vs-M-$ZnIn_2S_4$	80 mL of 10 vol% of lactic acid aqueous solution (pH=1.68)	300 W xenon lamp (λ=320−780 nm)	6.88	63.87 at 420 nm	every 5 h reaction for twenty cycles	17
$AgIn_5S_8$/$ZnIn_2S_4$	100 mL of aqueous solution containing 0.25 M Na_2SO_3 and 0.25 M Na_2S, 2% Pt	300 W xenon lamp with a 420 nm cutoff filter	0.949	/	every 5 h reaction for three cycles	18
UiO-66@$ZnIn_2S_4$	100 mL of aqueous solution containing15 mL of triethanolamine	300 W xenon lamp with a 400 nm cutoff filter	3.06	9.84 at 420 nm	every 4h reaction for four cycles	19
MoS_2/$ZnIn_2S_4$	100 mL of aqueous solution containing 0.25 M Na_2SO_3 and 0.35 M Na_2S	300 W xenon lamp with a 420 nm cutoff filter	3.89	/	every 4h reaction for three cycles	20
CoNi-$ZnIn_2S_4$	100 mL of aqueous solution containing 0.1M of ascorbic acid	300 W xenon lamp with a 420 nm cutoff filter	3.34	2.15 at 420 nm	every 4 h reaction for four cycles	21

Table S4 Apparent quantum efficiencies of ZTNs-Ce20 at different monochromatic light irradiation.

**Wavelength (nm)**	**H_2 amount (μmol)**	**Irradiation area (cm^2)**	**Light intensity (mW/cm^2)**	**Reaction time (h)**	**AQE (%)**
380	337.1	12.57	17.9	4	6.56
400	312.3	12.57	22.3	4	4.64
420	222.8	12.57	24.7	4	2.84
450	118.4	12.57	26.8	4	1.29

1. S. B. Wang, B. Y. Guan, X. Wang, X. W. Lou, Formation of Hierarchical Co_9S_8@$ZnIn_2S_4$ Heterostructured Cages as an Efficient Photocatalyst for Hydrogen Evolution, *J. Am. Chem. Soc*. **2018**, *140*, 15145-15148.
2. X.Y. Zhang, S.P. Ding, X.X. Luo, C. Huang, T.A. Dong, Y. Yang, N.K. Liu, J.C. Hu, Engineering amorphous red phosphorus onto $ZnIn_2S_4$ hollow microspheres with enhanced photocatalytic activity, *Mater. Lett.* **2018**, *232*, 78-81.
3. H. Li, Z.H. Chen, L. Zhao, G.D. Yang, Synthesis of TiO_2@$ZnIn_2S_4$ hollow nanospheres with enhanced photocatalytic hydrogen evolution, *Rare Metals.* **2019**, *38*, 420-427.
4. E.H. Zhang, Q.H. Zhu, J.H. Huang, J. Liu, G.Q. Tan, C.J. Sun, T. Li, S. Liu, Y.M. Li, H.Z. Wang, X.D. Wan, Z.H. Wen, F.T. Fan, J.T. Zhang, K. Ariga, Visually resolving the direct Z-scheme heterojunction in CdS@$ZnIn_2S_4$ hollow cubes for photocatalytic evolution of H_2 and H_2O_2 from pure water, *Appl. Catal. B: Environ*. **2021**, *293*, 120213.
5. K. Wu, L. Mao, X.Q. Gu, X.Y. Cai, Y.L. Zhao, Efficient charge separation in hierarchical NiS@$ZnIn_2S_4$ hollow nanospheres for photocatalytic water splitting, *Chin. Chem. Lett.* **2021**.
6. Y.G. Chao, P. Zhou, J.P. Lai, W.Y. Zhang, H.W. Yang, S.Y. Lu, H. Chen, K. Yin, M.G. Li, L. Tao, C.S. Shang, M.P. Tong, and S.J. Guo, $Ni_{1-x}Co_xSe_2$-C/$ZnIn_2S_4$ Hybrid Nanocages with Strong 2D/2D Hetero-Interface Interaction Enable Efficient H_2-Releasing Photocatalysis, *Adv. Funct. Mater.* **2021**, *31*, 2100923.
7. H. Liu, J. Zhang, D. Ao, Construction of heterostructured $ZnIn_2S_4$@NH_2-MIL-125(Ti) nanocomposites for visible-light-driven H_2 production, *Appl. Catal. B: Environ*. **2018**, *221*, 433-442.
8. X. L. Li, X. J. Wang, J. Y. Zhu, Y. P. Li, J. Zhao, F. T. Li, Fabrication of two-dimensional

$Ni_2P/ZnIn_2S_4$ heterostructures for enhanced photocatalytic hydrogen evolution, *Chem. Eng. J.* **2018**, *353*, 15-24.

9. G. C. Zuo, Y.T. Wang, W. L. Teo, A.M. Xie, Y. Guo, Y. X. Dai, W.Q. Zhou, D. Jana, Q.M. Xian, W. Dong, Y.L. Zhao, Ultrathin $ZnIn_2S_4$ Nanosheets Anchored on Ti_3C_2TX MXene for Photocatalytic H_2 Evolution, *Angew. Chem. Int. Ed*. **2020**, *59*, 11287-11292.
10. Y. Xia, Q. Li, K.L. Lv, D.G. Tang, M. Li, Superiority of graphene over carbon analogs for enhanced photocatalytic H2-production activity of $ZnIn_2S_4$, *Appl. Catal. B: Environ*. **2017**, *206*, 344-352.
11. Q. W. Liu, M. D. Wang, Y. S. He, X. X. Wang, W. Y. Su, Photochemical route for synthesizing Co-P alloy decorated $ZnIn_2S_4$ with enhanced photocatalytic H_2 production activity under visible light irradiation, *Nanoscale* **2018**, *10*, 19100-19106.
12. B. Lin, H. Li, H. An, W.B. Hao, J.J. Wei, Y.Z. Dai, C.S. Ma, G.D. Yang, Preparation of 2D/2D g-C_3N_4 nanosheet@$ZnIn_2S_4$ nanoleaf heterojunctions with well-designed high-speed charge transfer nanochannels towards high-efficiency photocatalytic hydrogen evolution, *Appl. Catal. B: Environ*. **2018**, *220*, 542-552.
13. S.B. Wang, Y. Wang, S.L. Zhang, S.Q. Zang, X.W. Lou, Supporting Ultrathin $ZnIn_2S_4$ Nanosheets on Co/N-doped Graphitic Carbon Nanocages for Efficient Photocatalytic H_2 Generation, *Adv. Mater*. **2019**, *31*, 1903404.
14. G.P. Zhang, D.Y. Chen, N.J. Li, Q.F. Xu, H. Li, J.H. He, J.M. Lu, Construction of Hierarchical Hollow $Co_9S_8/ZnIn_2S_4$ Tubular Heterostructures for Highly Efficient Solar Energy Conversion and Environmental Remediation, *Angew. Chem. Int. Ed.* **2020**, *59*, 8255-8261.
15. R.S. Zhu, F. Tian, S.N. Che, G. Cao, F. Ouyang, The photocatalytic performance of modified $ZnIn_2S_4$ with graphene and La for hydrogen generation under visible light, *Renew. Energy*. **2017**, *113*, 1503-1514.
16. P.F. Tan, A.Q. Zhu, L.L. Qiao, W.X. Zeng, Y.J. Ma, H.G. Dong, J.P. Xie, J. Pan, Constructing a direct Z-scheme photocatalytic system based on 2D/2D WO3/ZnIn2S4 nanocomposite for efficient hydrogen evolution under visible light. *Inorg. Chem. Front*. **2019**, *6*, 929-939.
17. S.Q. Zhang, X. Liu, C.B. Liu, S.L. Luo, L.L. Wang, T. Cai, Y.X. Zeng, J.L. Yuan, W.Y. Dong, Y. Pei, Y.T. Liu, MoS_2 Quantum Dot Growth Induced by S Vacancies in a $ZnIn_2S_4$ Monolayer:

Atomic-Level Heterostructure for Photocatalytic Hydrogen Production, *ACS Nano*. **2018**, *12*, 751-758.

18. Z.J. Guan, Z.Q. Xu, Q.Y. Li, P. Wang, G.Q. Li, J.J. Yang, $AgIn_5S_8$ nanoparticles anchored on 2D layered $ZnIn_2S_4$ to form 0D/2D heterojunction for enhanced visible-light photocatalytic hydrogen evolution, *Appl. Catal. B: Environ*. **2018**, *227*, 512-518.
19. X.X. Peng, L. Ye, Y.C. Ding, L.C. Yi, C. Zhang, Z.H. Wen, Nanohybrid Photocatalysts with $ZnIn_2S_4$ Nanosheets Encapsulated UiO-66 Octahedral Nanoparticles for Visible-Light-Driven Hydrogen Generation, *Appl. Catal. B: Environ*. **2020**, *260*, 118152.
20. Z.Z. Zhang, L. Huang, J.J. Zhang, F.J. Wang, Y.Y. Xie, X.T. Shang, Y.Y. Gu, H.B. Zhao, X.X. Wang, *In situ* constructing interfacial contact $MoS_2/ZnIn_2S_4$ heterostructure for enhancing solar photocatalytic hydrogen evolution, *Appl. Catal. B: Environ*. **2018**, *233*, 112-119.
21. Z.J. Li, X.H. Wang, W.L. Tian, A.L. Meng, L.N. Yang, CoNi bimetal co-catalyst modifying Hierarchical $ZnIn_2S_4$ nanosheet-based microspheres noble-metal-free photocatalyst for efficient visible-light-driven photocatalytic hydrogen production, *ACS Sustainable Chem. Eng*. **2019**, *7*, 20190-20201.
